# The analysis of novel microRNA mimic sequences in cancer cells reveals lack of specificity in stem-loop RT-qPCR-based microRNA detection

**DOI:** 10.1186/s13104-017-2930-0

**Published:** 2017-11-17

**Authors:** Patrick Winata, Marissa Williams, Eileen McGowan, Najah Nassif, Nico van Zandwijk, Glen Reid

**Affiliations:** 1 Asbestos Diseases Research Institute, Sydney, NSW 2139 Australia; 2 School of Medicine, University of Sydney, Sydney, NSW 2006 Australia; 3 University of Technology Sydney, Sydney, NSW 2007 Australia

**Keywords:** MicroRNA, Mimic, RT-qPCR, TargomiR, MesomiR

## Abstract

**Objective:**

MicroRNAs are frequently downregulated in cancer, and restoring expression has tumour suppressive activity in tumour cells. Our recent phase I clinical trial investigated microRNA-based therapy in patients with malignant pleural mesothelioma. Treatment with TargomiRs, microRNA mimics with novel sequence packaged in EGFR antibody-targeted bacterial minicells, revealed clear signs of clinical activity. In order to detect delivery of microRNA mimics to tumour cells in future clinical trials, we tested hydrolysis probe-based assays specific for the sequence of the novel mimics in transfected mesothelioma cell lines using RT-qPCR.

**Results:**

The custom assays efficiently and specifically amplified the consensus mimics. However, we found that these assays gave a signal when total RNA from untransfected and control mimic-transfected cells were used as templates. Further investigation revealed that the reverse transcription step using stem-loop primers appeared to introduce substantial non-specific amplification with either total RNA or synthetic RNA templates. This suggests that reverse transcription using stem-loop primers suffers from an intrinsic lack of specificity for the detection of highly similar microRNAs in the same family, especially when analysing total RNA. These results suggest that RT-qPCR is unlikely to be an effective means to detect delivery of microRNA mimic-based drugs to tumour cells in patients.

## Introduction

MicroRNAs are short non-coding RNAs that play a role in post-transcriptional regulation of gene expression [1, 2]. In several human cancers, microRNAs with tumour-suppressive properties are down-regulated [3], and the potential therapeutic application of re-expressing these microRNAs has drawn significant attention in recent years [4]. A well-studied example of microRNAs dysregulated in cancer is the miR-15/16 family. This was first demonstrated in chronic lymphocytic leukaemia where there is frequent loss of miR-15a/16-1 expression due to deletion of the 13q14 region [5]. Subsequent studies confirmed the importance of miR-16 in suppressing cancer-associated genes such as BCL2 [6]. A duplication of miR-15a/16-1—miR-15b/16-2—is located at locus 3q26 [6]. Another family member, miR-195, is located on chromosome 17 and has been linked to targeting of Raf-1 and Ccnd1 [7]. Recent studies have shown that this is suppressed in solid tumours including non-small cell lung cancer (NSCLC) [8], prostate cancer [9] and malignant pleural mesothelioma (MPM) [10]. In these and other tumour types, increasing the levels of the miR-15/16 family by transfecting cells with microRNA mimics resulted in growth inhibitory effects in vitro.

Due to sequence similarity at positions 1–7, several authors have proposed that miR-103 and miR-107 together with the miR-15 members, form the larger miR-15/107 group [11]. Our own studies led to the development of a series novel mimics based on the consensus sequence derived from members of the miR-15/107 group [12]. These mimics later became the microRNA component of targeted microRNA-loaded minicells (TargomiRs) and a phase I trial was recently completed [13, 14]. As TargomiRs contain a consensus mimic, this provides the possibility to specifically detect the non-natural sequences. Such an assay would be of use in future trials to confirm delivery of microRNA mimics into the tumour cells of patients.

In this study we employed an RT-qPCR method that uses a microRNA-specific stem-loop reverse transcription (RT) primer and a hydrolysis probe-based qPCR assay to specifically amplify microRNAs [15]. Using this method, *let-7* microRNA family members, some differing by only one nucleotide, could be specifically detected [15], suggesting that the same approach would allow us to discriminate the consensus sequence mimics from the natural microRNAs on which their sequence is based. Here we report that despite high specificity of detection and quantification for the consensus mimics using synthetic RNA templates, there is a lack of specificity of the method in the detection of the novel microRNA in transfected cells. This suggests that alternative methods will be required to track the delivery of microRNA mimics in patients.

## Main text

### Methods

#### Cell lines and culture

The human MPM cell line MSTO-211H was purchased from ATCC (Rockville, USA) and VMC23 was kindly provided by A/Prof Michael Grusch at the Medical University of Vienna, Austria. Cells were cultured in RPMI medium 1640 with 10% foetal bovine serum (FBS) (both Life Technologies, Carlsbad, CA, USA) at 37 °C with 5% CO_2_.

#### Synthetic microRNA template and mimics

Synthetic RNAs with sequences corresponding to the guide strands of hsa-miR-15a, hsa-miR-15b, hsa-miR-16, conmiR-15/107.2 and conmiR-15/107.4 were purchased from Integrated DNA Technologies (IDT, Iowa, USA). The microRNA mimics detailed in Table 1 were synthesised by GenePharma (Shanghai, China). All templates and mimics were reconstituted in nuclease-free water.

**Table 1:**
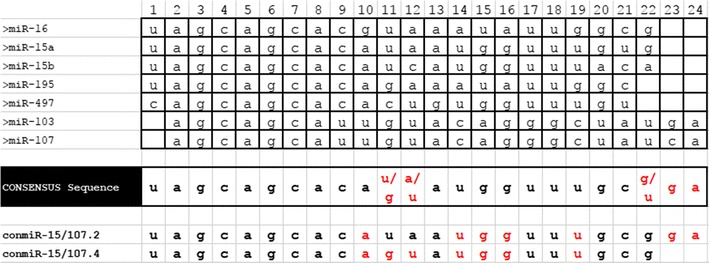
The list of miR-15/107 group members that were used to generate the consensus sequence

Nucleotide position is indicated above and in red are the positions for which there was no base that predominated. The consensus sequence was used to generate consensus mimics, of which conmiR-15/107.2 and 107.4 were most active in in vitro studies, and for which custom TaqMan assays were designed

#### Reverse transfection

Reverse transfection of microRNA mimics and controls was performed using Lipofectamine RNAiMAX (Life Technologies, Carlsbad, CA, USA) as described [10]. Cells (9 × 10^4^ per well in 6-well plates) were transfected with miR-16, conmiR-15/107.2, conmiR-15/107.4 or negative control at a final concentration of 5 nM, and were harvested 48 h post transfection for RNA isolation.

#### RNA isolation

Total RNA was extracted from cells using TRIzol (Life Technologies, Carlsbad, USA) as described [10], and concentration and sample quality assessed using a Nanophotometer (Implen, Munich, Germany). RNA was aliquoted and stored at − 80 °C until further use.

#### RT-qPCR

TaqMan assays for hsa-miR-15a, hsa-miR-15b hsa-miR-16 and custom assays for conmiR-15/107.2 and conmiR-15/107.4 were purchased from Life Technologies. RNA was reverse transcribed using the TaqMan MicroRNA Reverse Transcription kit (Life Technologies, Carlsbad, USA). The reactions were carried out on a MultiGene Thermocycler (LabNet International Inc., Edison, NJ, USA) with the following parameters: 30 min at 16 °C, 30 min at 42 °C, 5 min at 85 °C and immediately cooled at 4 °C. The resulting cDNAs were diluted 1 in 10 with nuclease-free water. Following the RT reaction, qPCR was performed immediately using KAPA Probe Master mix (Kapa Biosystems, Wilmington, Massachusetts, USA) under the following cycling conditions: 95 °C for 20 s, and then 40 cycles of 95 °C for 1 s and 60 °C for 20 s.

#### RT-qPCR assay efficiency and specificity calculation

The efficiency and specific of the RT-qPCR assays were assessed using formulae described by Pfaffl et al. [16] with minor modifications. Efficiency was determined by reverse transcribing 20 ng of synthetic RNA templates, diluting as described above, and then diluting further to generate a two-fold dilution series to produce a standard curve. The qPCR results were used to calculating efficiency with the formula:

$${\text{E}} = \left({\left({10^{{(-1/{\text{slope}})}} } \right) - 1} \right) \times 100\%$$

**Fig. 1:**
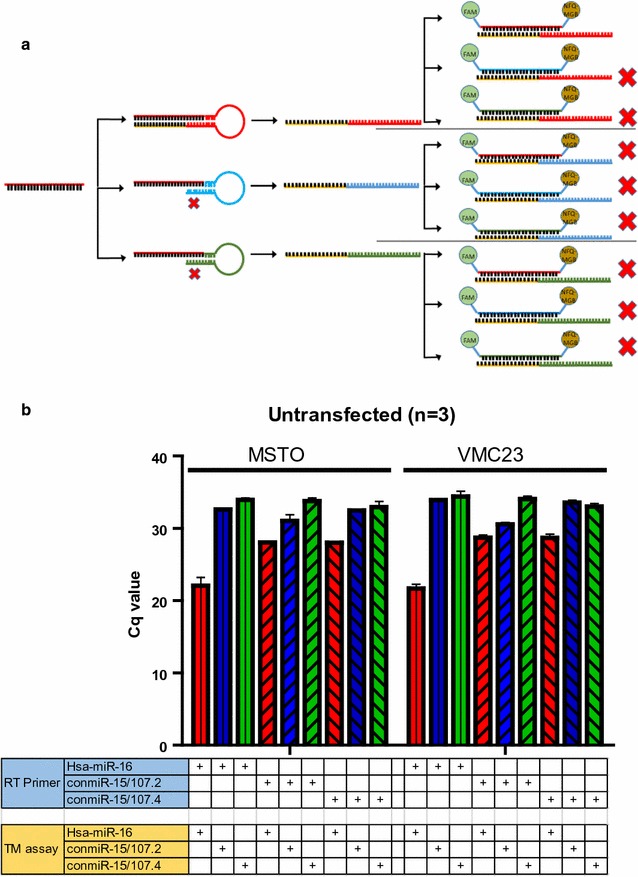
Determining specificity of assays specific for consensus mimics with total RNA as template. **a** Experimental design, in which each RT primer is combined with each TaqMan assay; only the specific combination of RT primer and TaqMan assay for miR-16 (all coloured red) should produce a signal in RNA from untransfected cells. **b** RT-qPCR data produced with each RT primer/TaqMan assay combination using RNA isolated from untransfected MSTO and VMC23 cells. Data are raw Cq values and the mean ± SD of three replicate experiments

To assess specificity, we performed RT reactions with each synthetic RNA template (20 ng) using three different stem-loop RT primers followed by qPCR using three different TaqMan assays, resulting in a matrix in which only one combination is specific to the template (Fig. 1). To further assess assay performance, we performed RT-qPCR using 100 ng of total RNA isolated from cells. We used the Cq values from each RT primer and TaqMan assay combination to calculate specificity using the formula:

$${\text{R }} = { 1}/ 2^{{\Delta {\text{Cq}}}} \times { 1}00\%$$

### Results

We first determined the efficiency and specificity of the assays to their respective synthetic RNA template. All three assays were able to efficiently amplify their respective microRNA species: hsa-miR-16 (87.3%), conmiR-15/107.2 (81.1%) and conmiR-15/107.4 (75.3%). We then tested the specificity of each assay using the experimental scheme described in Fig. 1a. Here, each synthetic RNA template was separately reverse transcribed with three RT primers, and each cDNA then used as template for qPCR using each TaqMan assay. For miR-16 and conmiR-15/107.4, only the specific RT primer and TaqMan assay combination led to discernible amplification. In contrast, the experiments with the conmiR-15/107-2 template indicated cross-reactivity with the RT primer specific for conmiR-15/107.4.

The cross reactivity of the custom assay was further investigated using total RNA from cell lines as template. First, we used total RNA from untransfected cells in which only miR-16 was present, and applied a matrix similar to that in Fig. 1a. As expected, the miR-16-specific RT primer and TaqMan assay resulted in a Cq value in the early 20 s, as previously observed [10]. However, there were signals with a Cq of less than 30 when cDNA generated with the RT primers for conmiR-15/107.2 and conmiR-15/107.4 was amplified with the miR-16-specific TaqMan assay (Fig. 1b). In addition, the conmiR-15/107.2-specific RT primer and TaqMan assay produced a Cq value of 30.5.

**Fig. 2:**
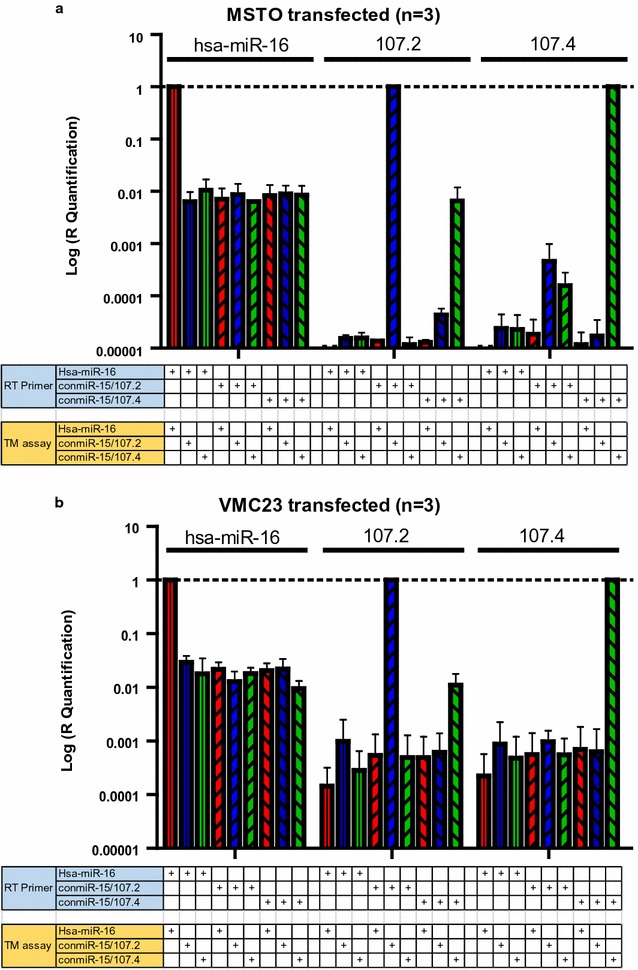
RT-qPCR-based detection of consensus mimics in transfected cell lines. The MPM cell lines MSTO (**a**) and VMC23 (**b**) were transfected with microRNA mimics as indicated above the figures, at a final concentration of 5 nM. Total RNA from each transfection was converted to cDNA using the indicated RT primer and qPCR carried out with the specified TaqMan assay. Data are normalised relative to the values obtained using the TaqMan assay specific for the mimic with which the cell line was transfected, and are the mean ± SD of three replicate experiments

To further test this apparent cross reactivity between microRNA assays, total RNA from mimic transfected cells was used as template (Fig. 2). The transfection of miR-16 mimics into the cells resulted in a 10- to 100-fold increase in miR-16 levels compared with cells transfected with conmiR-15/107.2 or conmiR-15/107.4, which already have substantial miR-16 expression (Fig. 2). In conmiR-15/107.2 transfected cells we observed a much larger increase in amplification compared with cells transfected with miR-16 or conmiR-15/107.4 for both lines. However, there was also a substantial signal using the conmiR-15/107.4 assay. Similarly, there was a non-specific signal detected in the RNA from conmiR-15/107.4-transfected cells when the conmiR-15/107.2 RT primer and assay were used.

### Discussion

Using microRNAs as therapeutic agents has long held promise for cancer treatment. Recently, two phase I trials were carried out to test the safety and optimal dose of microRNAs, with one showing early signs of activity [13]. In the next stage efficacy will be tested and an important part of demonstrating activity is to confirm the delivery of microRNA-based drugs to cells within the tumour. RT-qPCR is an attractive option to detect and quantify microRNAs due to its speed, low cost and purported specificity [17]. We chose to use a two-step RT-qPCR platform in an attempt to specifically detect our novel microRNA mimic sequences [10]. This method, which employs microRNA-specific stem-loop RT primers, gained prominence as it was demonstrated to specifically amplify a large number of microRNAs, and was able to discriminate between the highly similar members of the *let-7* family [15]. Our results for the most part confirm these findings. When using synthetic RNA templates, we found that each assay was highly specific for its intended template when the RT primer and hydrolysis probe and PCR primers from the same TaqMan assay were used.

To further investigate potential cross reactivity, we combined cDNA synthesised with the RT primer from one assay with qPCR detection using the hydrolysis primers and probe from another and found some low level cross reactivity. This was most apparent in experiments using the custom assays, in which the conmiR-15/107.4 assay was able to detect conmiR-15/107.2 template at a Cq only four cycles later than the conmiR-15/107.2 specific assay. Combining the conmiR-15/107.2 RT primer with the conmiR-15/107.4-specific qPCR assay did not produce the same cross reactivity, suggesting that the RT step introduces cross-reactivity. Interestingly, in the original Chen study, the authors did not use the combination of stem-loop primer specific for one microRNA with qPCR specific for another that we adopted here, although they did identify mispriming due to G:T mismatches during the RT step as the most likely cause of cross reactivity when detecting the most similar family members (let-7a and let-7c) [15]. In our study, there was greater cross reactivity evident when total RNA from cell lines was used as template for RT-qPCR. We ‘detected’ both conmiR-15/107.2 and conmiR-15/107.4 in total RNA from untransfected MPM cell lines, which can only be the result of non-specific reverse transcription and amplification of related endogenous microRNAs. The underlying microRNAs responsible for these results are unknown at present, but due to the ubiquitously high expression of the miR-15/16 family members in most cell lines, it is likely that they contribute at least in part.

Based on our data showing cross reactivity of the consensus mimic-specific TaqMan assays for endogenous microRNAs, it is interesting to consider how this might impact on the detection of these mimics in tumour biopsies in future clinical studies. Our phase I trial of TargomiRs revealed a maximum tolerated dose of 5 × 10^9^ minicells, which is equivalent to 1.5 µg mimic [13]. Considering that RT-qPCR for the consensus mimics using total RNA from untransfected cell lines gave Cq values of around 30, even 100% delivery of this dose to the tumour is unlikely to enable detection above this level in total RNA from a tumour biopsy. This is also complicated by the fact that although suppressed, the expression of miR-16 and related microRNAs remains relatively high in tumours and is ubiquitously expressed in normal tissue. This is in contrast to the case of miR-34a mimics, which in preclinical studies were shown to increase in xenograft tumour tissue following tail-vein injection [18]. The high abundance of miR-16 in red blood cells and plasma [19] would also make RT-qPCR-based determination of pharmacokinetics very difficult. This is in contrast to the observations related to the miR-34a trial, in which the increase in miR-34a in blood could be readily quantified by RT-qPCR due to the higher doses used and the lower abundance of miR-34a in the blood [20].

In conclusion, we have found that an RT-qPCR-based approach to the detection of novel microRNA mimics related to the miR-15 family is able to distinguish their sequences from endogenous microRNAs, but has difficulties when trying to discriminate between microRNAs populations in total RNA. Therefore, it is likely that determination of microRNA delivery in future studies will need to employ a sequencing-based approach.

#### Limitations

Our results indicate a low-level cross reactivity of TaqMan assays specific for related microRNAs. Although the RT step would appear to be responsible, the precise mechanism responsible for this observation remains unclear and would require cloning and sequencing of qPCR products. In addition, our study only used miR-16 as a wild–type microRNA species in the comparison with two consensus sequences, and other microRNAs, especially miR-15a and 15b could also potentially contribute to cross reactivity.

## Abbreviations

RT: reverse transcription
RT-qPCR: reverse transcription polymerase chain reaction
Cq: quantification cycle
NSCLC: non-small cell lung cancer
MPM: malignant pleural mesothelioma
